# Supplementary material for: Multi‐dataset identification of innovative feature genes and molecular mechanisms in keratoconus
Source: J Cell Mol Med. 2024 Sep 19;28(18):e70079. doi: 10.1111/jcmm.70079 (PMC11412914; doi:10.1111/jcmm.70079)
Supplement: Supplementary file 2 — Material S2. [file JCMM-28-e70079-s002.zip › 7.GSEA analysis/2.ATOH7/gsea_report_for_h_1678852790847.html]

Report for h 1678852790847 [GSEA]

| GS  follow link to MSigDB | GS DETAILS | SIZE | ES | NES | NOM p-val | FDR q-val | FWER p-val | RANK AT MAX | LEADING EDGE || 1 | KEGG\_BASAL\_CELL\_CARCINOMA | Details ... | 55 | 0.62 | 1.81 | 0.000 | 0.230 | 0.143 | 6644 | tags=44%, list=19%, signal=54% |
| 2 | KEGG\_ADIPOCYTOKINE\_SIGNALING\_PATHWAY | Details ... | 66 | 0.49 | 1.73 | 0.006 | 0.344 | 0.320 | 8689 | tags=33%, list=25%, signal=44% |
| 3 | KEGG\_ALZHEIMERS\_DISEASE | Details ... | 144 | 0.33 | 1.72 | 0.008 | 0.243 | 0.330 | 3497 | tags=7%, list=10%, signal=8% |
| 4 | KEGG\_PARKINSONS\_DISEASE | Details ... | 98 | 0.40 | 1.68 | 0.026 | 0.278 | 0.424 | 1278 | tags=4%, list=4%, signal=4% |
| 5 | KEGG\_MELANOGENESIS | Details ... | 101 | 0.45 | 1.68 | 0.000 | 0.224 | 0.424 | 3497 | tags=23%, list=10%, signal=25% |
| 6 | KEGG\_EPITHELIAL\_CELL\_SIGNALING\_IN\_HELICOBACTER\_PYLORI\_INFECTION | Details ... | 66 | 0.49 | 1.66 | 0.027 | 0.227 | 0.473 | 6672 | tags=26%, list=19%, signal=32% |
| 7 | KEGG\_TYPE\_II\_DIABETES\_MELLITUS | Details ... | 47 | 0.52 | 1.60 | 0.031 | 0.359 | 0.608 | 1752 | tags=19%, list=5%, signal=20% |
| 8 | KEGG\_GLYCOLYSIS\_GLUCONEOGENESIS | Details ... | 61 | 0.43 | 1.59 | 0.023 | 0.338 | 0.627 | 11206 | tags=49%, list=32%, signal=72% |
| 9 | KEGG\_PROSTATE\_CANCER | Details ... | 89 | 0.38 | 1.58 | 0.014 | 0.314 | 0.637 | 1098 | tags=9%, list=3%, signal=9% |
| 10 | KEGG\_NITROGEN\_METABOLISM | Details ... | 23 | 0.60 | 1.58 | 0.037 | 0.284 | 0.638 | 7210 | tags=48%, list=21%, signal=60% |
| 11 | KEGG\_WNT\_SIGNALING\_PATHWAY | Details ... | 149 | 0.37 | 1.57 | 0.008 | 0.288 | 0.670 | 3424 | tags=17%, list=10%, signal=19% |
| 12 | KEGG\_INOSITOL\_PHOSPHATE\_METABOLISM | Details ... | 54 | 0.41 | 1.57 | 0.043 | 0.276 | 0.676 | 10067 | tags=37%, list=29%, signal=52% |
| 13 | KEGG\_BUTANOATE\_METABOLISM | Details ... | 34 | 0.53 | 1.56 | 0.030 | 0.267 | 0.681 | 4303 | tags=21%, list=12%, signal=23% |
| 14 | KEGG\_GLYCOSAMINOGLYCAN\_BIOSYNTHESIS\_CHONDROITIN\_SULFATE | Details ... | 22 | 0.60 | 1.56 | 0.039 | 0.257 | 0.694 | 9120 | tags=50%, list=26%, signal=68% |
| 15 | KEGG\_PPAR\_SIGNALING\_PATHWAY | Details ... | 69 | 0.47 | 1.53 | 0.023 | 0.285 | 0.733 | 3084 | tags=23%, list=9%, signal=25% |
| 16 | KEGG\_INSULIN\_SIGNALING\_PATHWAY | Details ... | 136 | 0.35 | 1.52 | 0.048 | 0.285 | 0.743 | 6929 | tags=19%, list=20%, signal=24% |
| 17 | KEGG\_ARRHYTHMOGENIC\_RIGHT\_VENTRICULAR\_CARDIOMYOPATHY\_ARVC | Details ... | 74 | 0.48 | 1.52 | 0.032 | 0.275 | 0.746 | 6683 | tags=35%, list=19%, signal=43% |
| 18 | KEGG\_VIBRIO\_CHOLERAE\_INFECTION | Details ... | 51 | 0.36 | 1.52 | 0.028 | 0.262 | 0.747 | 5651 | tags=16%, list=16%, signal=19% |
| 19 | KEGG\_ACUTE\_MYELOID\_LEUKEMIA | Details ... | 57 | 0.36 | 1.52 | 0.036 | 0.249 | 0.751 | 7693 | tags=26%, list=22%, signal=34% |
| 20 | KEGG\_TYROSINE\_METABOLISM | Details ... | 40 | 0.47 | 1.51 | 0.017 | 0.245 | 0.754 | 3743 | tags=28%, list=11%, signal=31% |
| 21 | KEGG\_SPHINGOLIPID\_METABOLISM | Details ... | 35 | 0.43 | 1.49 | 0.049 | 0.274 | 0.793 | 8626 | tags=34%, list=25%, signal=46% |
| 22 | KEGG\_AMYOTROPHIC\_LATERAL\_SCLEROSIS\_ALS | Details ... | 53 | 0.43 | 1.49 | 0.051 | 0.267 | 0.797 | 7669 | tags=25%, list=22%, signal=31% |
| 23 | KEGG\_AMINO\_SUGAR\_AND\_NUCLEOTIDE\_SUGAR\_METABOLISM | Details ... | 42 | 0.41 | 1.48 | 0.099 | 0.270 | 0.813 | 10220 | tags=31%, list=29%, signal=44% |
| 24 | KEGG\_FC\_GAMMA\_R\_MEDIATED\_PHAGOCYTOSIS | Details ... | 92 | 0.36 | 1.48 | 0.064 | 0.265 | 0.816 | 4910 | tags=15%, list=14%, signal=18% |
| 25 | KEGG\_PRIMARY\_BILE\_ACID\_BIOSYNTHESIS | Details ... | 16 | 0.65 | 1.47 | 0.046 | 0.269 | 0.822 | 5702 | tags=56%, list=16%, signal=67% |
| 26 | KEGG\_ADHERENS\_JUNCTION | Details ... | 68 | 0.39 | 1.47 | 0.105 | 0.264 | 0.827 | 6644 | tags=21%, list=19%, signal=25% |
| 27 | KEGG\_HEDGEHOG\_SIGNALING\_PATHWAY | Details ... | 55 | 0.46 | 1.45 | 0.016 | 0.275 | 0.843 | 4085 | tags=29%, list=12%, signal=33% |
| 28 | KEGG\_CHEMOKINE\_SIGNALING\_PATHWAY | Details ... | 183 | 0.41 | 1.45 | 0.042 | 0.275 | 0.849 | 5082 | tags=22%, list=15%, signal=26% |
| 29 | KEGG\_HUNTINGTONS\_DISEASE | Details ... | 158 | 0.26 | 1.44 | 0.067 | 0.291 | 0.866 | 15471 | tags=45%, list=45%, signal=81% |
| 30 | KEGG\_THYROID\_CANCER | Details ... | 29 | 0.42 | 1.43 | 0.068 | 0.286 | 0.869 | 4240 | tags=17%, list=12%, signal=20% |
| 31 | KEGG\_FC\_EPSILON\_RI\_SIGNALING\_PATHWAY | Details ... | 78 | 0.40 | 1.42 | 0.043 | 0.296 | 0.885 | 3338 | tags=18%, list=10%, signal=20% |
| 32 | KEGG\_VIRAL\_MYOCARDITIS | Details ... | 70 | 0.45 | 1.42 | 0.073 | 0.290 | 0.885 | 4808 | tags=24%, list=14%, signal=28% |
| 33 | KEGG\_ETHER\_LIPID\_METABOLISM | Details ... | 29 | 0.55 | 1.41 | 0.087 | 0.293 | 0.892 | 2876 | tags=24%, list=8%, signal=26% |
| 34 | KEGG\_PATHWAYS\_IN\_CANCER | Details ... | 322 | 0.33 | 1.41 | 0.054 | 0.288 | 0.892 | 8701 | tags=27%, list=25%, signal=36% |
| 35 | KEGG\_COLORECTAL\_CANCER | Details ... | 62 | 0.35 | 1.39 | 0.101 | 0.332 | 0.914 | 3059 | tags=10%, list=9%, signal=11% |
| 36 | KEGG\_TRYPTOPHAN\_METABOLISM | Details ... | 39 | 0.42 | 1.38 | 0.055 | 0.331 | 0.918 | 3694 | tags=18%, list=11%, signal=20% |
| 37 | KEGG\_ALANINE\_ASPARTATE\_AND\_GLUTAMATE\_METABOLISM | Details ... | 30 | 0.41 | 1.38 | 0.064 | 0.335 | 0.922 | 8279 | tags=33%, list=24%, signal=44% |
| 38 | KEGG\_NEUROTROPHIN\_SIGNALING\_PATHWAY | Details ... | 125 | 0.29 | 1.37 | 0.104 | 0.332 | 0.925 | 7693 | tags=20%, list=22%, signal=26% |
| 39 | KEGG\_MAPK\_SIGNALING\_PATHWAY | Details ... | 265 | 0.34 | 1.37 | 0.056 | 0.325 | 0.926 | 9481 | tags=33%, list=27%, signal=45% |
| 40 | KEGG\_GAP\_JUNCTION | Details ... | 86 | 0.30 | 1.35 | 0.035 | 0.355 | 0.932 | 5483 | tags=19%, list=16%, signal=22% |
| 41 | KEGG\_VASCULAR\_SMOOTH\_MUSCLE\_CONTRACTION | Details ... | 111 | 0.37 | 1.35 | 0.076 | 0.350 | 0.933 | 5666 | tags=23%, list=16%, signal=27% |
| 42 | KEGG\_INTESTINAL\_IMMUNE\_NETWORK\_FOR\_IGA\_PRODUCTION | Details ... | 48 | 0.51 | 1.35 | 0.088 | 0.349 | 0.937 | 5485 | tags=42%, list=16%, signal=49% |
| 43 | KEGG\_TIGHT\_JUNCTION | Details ... | 127 | 0.37 | 1.34 | 0.102 | 0.352 | 0.941 | 6460 | tags=23%, list=19%, signal=28% |
| 44 | KEGG\_ENDOCYTOSIS | Details ... | 179 | 0.25 | 1.34 | 0.119 | 0.348 | 0.942 | 9543 | tags=21%, list=27%, signal=28% |
| 45 | KEGG\_FRUCTOSE\_AND\_MANNOSE\_METABOLISM | Details ... | 32 | 0.48 | 1.34 | 0.173 | 0.341 | 0.942 | 11758 | tags=53%, list=34%, signal=80% |
| 46 | KEGG\_PROXIMAL\_TUBULE\_BICARBONATE\_RECLAMATION | Details ... | 23 | 0.44 | 1.34 | 0.064 | 0.334 | 0.942 | 7210 | tags=35%, list=21%, signal=44% |
| 47 | KEGG\_PURINE\_METABOLISM | Details ... | 151 | 0.31 | 1.33 | 0.131 | 0.338 | 0.946 | 7011 | tags=21%, list=20%, signal=26% |
| 48 | KEGG\_GLYCOSAMINOGLYCAN\_BIOSYNTHESIS\_KERATAN\_SULFATE | Details ... | 15 | 0.48 | 1.33 | 0.173 | 0.331 | 0.946 | 4606 | tags=33%, list=13%, signal=38% |
| 49 | KEGG\_CALCIUM\_SIGNALING\_PATHWAY | Details ... | 177 | 0.35 | 1.33 | 0.036 | 0.324 | 0.946 | 5961 | tags=27%, list=17%, signal=33% |
| 50 | KEGG\_CELL\_ADHESION\_MOLECULES\_CAMS | Details ... | 130 | 0.42 | 1.32 | 0.073 | 0.331 | 0.952 | 6846 | tags=32%, list=20%, signal=40% |
| 51 | KEGG\_PRION\_DISEASES |  | 35 | 0.46 | 1.31 | 0.128 | 0.357 | 0.962 | 6357 | tags=26%, list=18%, signal=31% |
| 52 | KEGG\_ENDOMETRIAL\_CANCER |  | 52 | 0.33 | 1.30 | 0.192 | 0.370 | 0.968 | 735 | tags=6%, list=2%, signal=6% |
| 53 | KEGG\_LYSINE\_DEGRADATION |  | 39 | 0.40 | 1.29 | 0.221 | 0.375 | 0.970 | 11422 | tags=44%, list=33%, signal=65% |
| 54 | KEGG\_GALACTOSE\_METABOLISM |  | 25 | 0.41 | 1.28 | 0.185 | 0.378 | 0.971 | 11405 | tags=44%, list=33%, signal=65% |
| 55 | KEGG\_LONG\_TERM\_DEPRESSION |  | 67 | 0.38 | 1.28 | 0.144 | 0.387 | 0.977 | 3125 | tags=16%, list=9%, signal=18% |
| 56 | KEGG\_ARGININE\_AND\_PROLINE\_METABOLISM |  | 52 | 0.37 | 1.28 | 0.156 | 0.380 | 0.977 | 975 | tags=8%, list=3%, signal=8% |
| 57 | KEGG\_RENAL\_CELL\_CARCINOMA |  | 66 | 0.42 | 1.28 | 0.166 | 0.374 | 0.977 | 10666 | tags=44%, list=31%, signal=63% |
| 58 | KEGG\_ANTIGEN\_PROCESSING\_AND\_PRESENTATION |  | 79 | 0.39 | 1.27 | 0.191 | 0.372 | 0.977 | 9775 | tags=33%, list=28%, signal=46% |
| 59 | KEGG\_AXON\_GUIDANCE |  | 128 | 0.33 | 1.27 | 0.126 | 0.377 | 0.978 | 5143 | tags=20%, list=15%, signal=23% |
| 60 | KEGG\_PHOSPHATIDYLINOSITOL\_SIGNALING\_SYSTEM |  | 76 | 0.30 | 1.26 | 0.166 | 0.382 | 0.979 | 10165 | tags=37%, list=29%, signal=52% |
| 61 | KEGG\_DNA\_REPLICATION |  | 36 | 0.38 | 1.26 | 0.244 | 0.377 | 0.979 | 12705 | tags=67%, list=37%, signal=105% |
| 62 | KEGG\_CELL\_CYCLE |  | 124 | 0.36 | 1.25 | 0.300 | 0.388 | 0.981 | 12208 | tags=44%, list=35%, signal=67% |
| 63 | KEGG\_ASTHMA |  | 29 | 0.57 | 1.25 | 0.217 | 0.383 | 0.981 | 4808 | tags=45%, list=14%, signal=52% |
| 64 | KEGG\_METABOLISM\_OF\_XENOBIOTICS\_BY\_CYTOCHROME\_P450 |  | 60 | 0.40 | 1.25 | 0.144 | 0.381 | 0.983 | 3650 | tags=25%, list=11%, signal=28% |
| 65 | KEGG\_STEROID\_HORMONE\_BIOSYNTHESIS |  | 46 | 0.41 | 1.25 | 0.106 | 0.379 | 0.984 | 6980 | tags=43%, list=20%, signal=54% |
| 66 | KEGG\_GLYCOSPHINGOLIPID\_BIOSYNTHESIS\_LACTO\_AND\_NEOLACTO\_SERIES |  | 26 | 0.43 | 1.24 | 0.167 | 0.374 | 0.984 | 462 | tags=12%, list=1%, signal=12% |
| 67 | KEGG\_REGULATION\_OF\_ACTIN\_CYTOSKELETON |  | 210 | 0.29 | 1.24 | 0.140 | 0.372 | 0.984 | 10210 | tags=32%, list=29%, signal=46% |
| 68 | KEGG\_VASOPRESSIN\_REGULATED\_WATER\_REABSORPTION |  | 44 | 0.35 | 1.24 | 0.236 | 0.375 | 0.984 | 2474 | tags=9%, list=7%, signal=10% |
| 69 | KEGG\_JAK\_STAT\_SIGNALING\_PATHWAY |  | 151 | 0.37 | 1.24 | 0.188 | 0.371 | 0.984 | 6380 | tags=28%, list=18%, signal=34% |
| 70 | KEGG\_APOPTOSIS |  | 87 | 0.31 | 1.23 | 0.177 | 0.369 | 0.984 | 7669 | tags=22%, list=22%, signal=28% |
| 71 | KEGG\_RETINOL\_METABOLISM |  | 55 | 0.38 | 1.23 | 0.082 | 0.367 | 0.984 | 3650 | tags=24%, list=11%, signal=26% |
| 72 | KEGG\_SYSTEMIC\_LUPUS\_ERYTHEMATOSUS |  | 56 | 0.51 | 1.23 | 0.248 | 0.363 | 0.984 | 5743 | tags=38%, list=17%, signal=45% |
| 73 | KEGG\_CYTOKINE\_CYTOKINE\_RECEPTOR\_INTERACTION |  | 256 | 0.41 | 1.23 | 0.195 | 0.359 | 0.984 | 6380 | tags=34%, list=18%, signal=41% |
| 74 | KEGG\_LONG\_TERM\_POTENTIATION |  | 70 | 0.29 | 1.22 | 0.179 | 0.376 | 0.986 | 4659 | tags=17%, list=13%, signal=20% |
| 75 | KEGG\_O\_GLYCAN\_BIOSYNTHESIS |  | 29 | 0.43 | 1.22 | 0.218 | 0.372 | 0.986 | 6205 | tags=24%, list=18%, signal=29% |
| 76 | KEGG\_DORSO\_VENTRAL\_AXIS\_FORMATION |  | 24 | 0.39 | 1.21 | 0.280 | 0.379 | 0.986 | 8070 | tags=29%, list=23%, signal=38% |
| 77 | KEGG\_DRUG\_METABOLISM\_CYTOCHROME\_P450 |  | 62 | 0.37 | 1.21 | 0.186 | 0.375 | 0.986 | 3650 | tags=21%, list=11%, signal=23% |
| 78 | KEGG\_BLADDER\_CANCER |  | 41 | 0.43 | 1.20 | 0.296 | 0.380 | 0.988 | 5247 | tags=17%, list=15%, signal=20% |
| 79 | KEGG\_RENIN\_ANGIOTENSIN\_SYSTEM |  | 17 | 0.52 | 1.20 | 0.207 | 0.376 | 0.988 | 5818 | tags=35%, list=17%, signal=42% |
| 80 | KEGG\_FOCAL\_ADHESION |  | 196 | 0.34 | 1.20 | 0.257 | 0.375 | 0.988 | 6683 | tags=23%, list=19%, signal=29% |
| 81 | KEGG\_MTOR\_SIGNALING\_PATHWAY |  | 51 | 0.34 | 1.19 | 0.267 | 0.388 | 0.989 | 2372 | tags=10%, list=7%, signal=11% |
| 82 | KEGG\_B\_CELL\_RECEPTOR\_SIGNALING\_PATHWAY |  | 75 | 0.29 | 1.18 | 0.214 | 0.394 | 0.989 | 3313 | tags=13%, list=10%, signal=15% |
| 83 | KEGG\_LEISHMANIA\_INFECTION |  | 71 | 0.42 | 1.17 | 0.271 | 0.407 | 0.991 | 8680 | tags=37%, list=25%, signal=49% |
| 84 | KEGG\_ALDOSTERONE\_REGULATED\_SODIUM\_REABSORPTION |  | 42 | 0.35 | 1.15 | 0.248 | 0.442 | 0.993 | 5226 | tags=24%, list=15%, signal=28% |
| 85 | KEGG\_ALLOGRAFT\_REJECTION |  | 37 | 0.47 | 1.14 | 0.322 | 0.455 | 0.994 | 4808 | tags=32%, list=14%, signal=38% |
| 86 | KEGG\_CHRONIC\_MYELOID\_LEUKEMIA |  | 73 | 0.29 | 1.14 | 0.273 | 0.450 | 0.994 | 8997 | tags=23%, list=26%, signal=31% |
| 87 | KEGG\_PATHOGENIC\_ESCHERICHIA\_COLI\_INFECTION |  | 54 | 0.26 | 1.13 | 0.326 | 0.455 | 0.995 | 2137 | tags=6%, list=6%, signal=6% |
| 88 | KEGG\_ARACHIDONIC\_ACID\_METABOLISM |  | 57 | 0.34 | 1.12 | 0.256 | 0.461 | 0.996 | 2876 | tags=18%, list=8%, signal=19% |
| 89 | KEGG\_LYSOSOME |  | 117 | 0.21 | 1.12 | 0.350 | 0.462 | 0.996 | 14954 | tags=49%, list=43%, signal=85% |
| 90 | KEGG\_N\_GLYCAN\_BIOSYNTHESIS |  | 46 | 0.34 | 1.11 | 0.403 | 0.474 | 0.997 | 4911 | tags=13%, list=14%, signal=15% |
| 91 | KEGG\_PYRUVATE\_METABOLISM |  | 40 | 0.29 | 1.10 | 0.355 | 0.487 | 0.997 | 8109 | tags=20%, list=23%, signal=26% |
| 92 | KEGG\_NON\_SMALL\_CELL\_LUNG\_CANCER |  | 54 | 0.24 | 1.10 | 0.314 | 0.483 | 0.997 | 2281 | tags=6%, list=7%, signal=6% |
| 93 | KEGG\_RIG\_I\_LIKE\_RECEPTOR\_SIGNALING\_PATHWAY |  | 64 | 0.30 | 1.10 | 0.349 | 0.480 | 0.997 | 7054 | tags=20%, list=20%, signal=25% |
| 94 | KEGG\_CARDIAC\_MUSCLE\_CONTRACTION |  | 72 | 0.29 | 1.10 | 0.256 | 0.476 | 0.997 | 4974 | tags=17%, list=14%, signal=19% |
| 95 | KEGG\_TYPE\_I\_DIABETES\_MELLITUS |  | 43 | 0.41 | 1.10 | 0.374 | 0.477 | 0.997 | 7427 | tags=37%, list=21%, signal=47% |
| 96 | KEGG\_GRAFT\_VERSUS\_HOST\_DISEASE |  | 39 | 0.44 | 1.09 | 0.372 | 0.478 | 0.998 | 8618 | tags=38%, list=25%, signal=51% |
| 97 | KEGG\_VEGF\_SIGNALING\_PATHWAY |  | 75 | 0.29 | 1.07 | 0.346 | 0.508 | 0.998 | 4743 | tags=15%, list=14%, signal=17% |
| 98 | KEGG\_GLYCEROPHOSPHOLIPID\_METABOLISM |  | 70 | 0.28 | 1.07 | 0.333 | 0.504 | 0.998 | 2876 | tags=10%, list=8%, signal=11% |
| 99 | KEGG\_P53\_SIGNALING\_PATHWAY |  | 66 | 0.30 | 1.07 | 0.390 | 0.511 | 0.998 | 9293 | tags=30%, list=27%, signal=41% |
| 100 | KEGG\_MELANOMA |  | 71 | 0.31 | 1.06 | 0.342 | 0.512 | 0.999 | 573 | tags=7%, list=2%, signal=7% |
| 101 | KEGG\_MATURITY\_ONSET\_DIABETES\_OF\_THE\_YOUNG |  | 25 | 0.40 | 1.06 | 0.354 | 0.509 | 0.999 | 2645 | tags=20%, list=8%, signal=22% |
| 102 | KEGG\_TOLL\_LIKE\_RECEPTOR\_SIGNALING\_PATHWAY |  | 96 | 0.31 | 1.06 | 0.381 | 0.506 | 0.999 | 6613 | tags=22%, list=19%, signal=27% |
| 103 | KEGG\_AUTOIMMUNE\_THYROID\_DISEASE |  | 48 | 0.39 | 1.06 | 0.368 | 0.501 | 0.999 | 4808 | tags=33%, list=14%, signal=39% |
| 104 | KEGG\_LEUKOCYTE\_TRANSENDOTHELIAL\_MIGRATION |  | 114 | 0.28 | 1.06 | 0.356 | 0.505 | 0.999 | 7020 | tags=23%, list=20%, signal=28% |
| 105 | KEGG\_PYRIMIDINE\_METABOLISM |  | 95 | 0.24 | 1.05 | 0.421 | 0.513 | 0.999 | 12507 | tags=39%, list=36%, signal=61% |
| 106 | KEGG\_NATURAL\_KILLER\_CELL\_MEDIATED\_CYTOTOXICITY |  | 125 | 0.28 | 1.05 | 0.375 | 0.509 | 0.999 | 6505 | tags=22%, list=19%, signal=26% |
| 107 | KEGG\_TASTE\_TRANSDUCTION |  | 52 | 0.36 | 1.04 | 0.370 | 0.514 | 0.999 | 1971 | tags=19%, list=6%, signal=20% |
| 108 | KEGG\_PANCREATIC\_CANCER |  | 69 | 0.24 | 1.04 | 0.421 | 0.513 | 0.999 | 4240 | tags=9%, list=12%, signal=10% |
| 109 | KEGG\_VALINE\_LEUCINE\_AND\_ISOLEUCINE\_DEGRADATION |  | 43 | 0.28 | 1.04 | 0.410 | 0.511 | 0.999 | 8935 | tags=23%, list=26%, signal=31% |
| 110 | KEGG\_NEUROACTIVE\_LIGAND\_RECEPTOR\_INTERACTION |  | 270 | 0.29 | 1.04 | 0.309 | 0.510 | 0.999 | 7454 | tags=33%, list=21%, signal=42% |
| 111 | KEGG\_HISTIDINE\_METABOLISM |  | 28 | 0.39 | 1.03 | 0.420 | 0.509 | 0.999 | 1890 | tags=14%, list=5%, signal=15% |
| 112 | KEGG\_MISMATCH\_REPAIR |  | 23 | 0.30 | 1.03 | 0.420 | 0.517 | 0.999 | 12501 | tags=61%, list=36%, signal=95% |
| 113 | KEGG\_GLUTATHIONE\_METABOLISM |  | 49 | 0.31 | 1.02 | 0.430 | 0.522 | 0.999 | 12446 | tags=43%, list=36%, signal=67% |
| 114 | KEGG\_PROPANOATE\_METABOLISM |  | 31 | 0.31 | 1.02 | 0.454 | 0.525 | 0.999 | 9500 | tags=26%, list=27%, signal=35% |
| 115 | KEGG\_SMALL\_CELL\_LUNG\_CANCER |  | 84 | 0.26 | 1.02 | 0.434 | 0.522 | 0.999 | 9539 | tags=27%, list=27%, signal=38% |
| 116 | KEGG\_REGULATION\_OF\_AUTOPHAGY |  | 30 | 0.32 | 1.00 | 0.436 | 0.557 | 0.999 | 8173 | tags=27%, list=24%, signal=35% |
| 117 | KEGG\_GNRH\_SIGNALING\_PATHWAY |  | 100 | 0.22 | 0.99 | 0.454 | 0.554 | 0.999 | 4240 | tags=13%, list=12%, signal=15% |
| 118 | KEGG\_DILATED\_CARDIOMYOPATHY |  | 90 | 0.27 | 0.99 | 0.479 | 0.561 | 0.999 | 6009 | tags=22%, list=17%, signal=27% |
| 119 | KEGG\_ERBB\_SIGNALING\_PATHWAY |  | 86 | 0.23 | 0.98 | 0.483 | 0.565 | 0.999 | 6166 | tags=15%, list=18%, signal=18% |
| 120 | KEGG\_HEMATOPOIETIC\_CELL\_LINEAGE |  | 87 | 0.32 | 0.97 | 0.488 | 0.579 | 0.999 | 8664 | tags=39%, list=25%, signal=52% |
| 121 | KEGG\_OXIDATIVE\_PHOSPHORYLATION |  | 98 | 0.22 | 0.96 | 0.504 | 0.592 | 1.000 | 15306 | tags=40%, list=44%, signal=71% |
| 122 | KEGG\_OLFACTORY\_TRANSDUCTION |  | 372 | 0.28 | 0.95 | 0.611 | 0.615 | 1.000 | 6319 | tags=27%, list=18%, signal=32% |
| 123 | KEGG\_PROGESTERONE\_MEDIATED\_OOCYTE\_MATURATION |  | 85 | 0.24 | 0.95 | 0.553 | 0.611 | 1.000 | 7052 | tags=18%, list=20%, signal=22% |
| 124 | KEGG\_GLYCEROLIPID\_METABOLISM |  | 43 | 0.27 | 0.95 | 0.573 | 0.608 | 1.000 | 3374 | tags=14%, list=10%, signal=15% |
| 125 | KEGG\_COMPLEMENT\_AND\_COAGULATION\_CASCADES |  | 69 | 0.30 | 0.94 | 0.558 | 0.611 | 1.000 | 6363 | tags=28%, list=18%, signal=34% |
| 126 | KEGG\_HYPERTROPHIC\_CARDIOMYOPATHY\_HCM |  | 83 | 0.27 | 0.94 | 0.546 | 0.609 | 1.000 | 6009 | tags=22%, list=17%, signal=26% |
| 127 | KEGG\_CITRATE\_CYCLE\_TCA\_CYCLE |  | 30 | 0.23 | 0.94 | 0.541 | 0.611 | 1.000 | 14104 | tags=43%, list=41%, signal=73% |
| 128 | KEGG\_NUCLEOTIDE\_EXCISION\_REPAIR |  | 43 | 0.21 | 0.91 | 0.570 | 0.648 | 1.000 | 13459 | tags=53%, list=39%, signal=87% |
| 129 | KEGG\_T\_CELL\_RECEPTOR\_SIGNALING\_PATHWAY |  | 107 | 0.25 | 0.90 | 0.610 | 0.676 | 1.000 | 6613 | tags=19%, list=19%, signal=23% |
| 130 | KEGG\_TGF\_BETA\_SIGNALING\_PATHWAY |  | 85 | 0.27 | 0.88 | 0.638 | 0.696 | 1.000 | 8610 | tags=29%, list=25%, signal=39% |
| 131 | KEGG\_PENTOSE\_PHOSPHATE\_PATHWAY |  | 27 | 0.23 | 0.88 | 0.654 | 0.696 | 1.000 | 4791 | tags=15%, list=14%, signal=17% |
| 132 | KEGG\_GLYCINE\_SERINE\_AND\_THREONINE\_METABOLISM |  | 31 | 0.26 | 0.88 | 0.720 | 0.693 | 1.000 | 6514 | tags=29%, list=19%, signal=36% |
| 133 | KEGG\_CYTOSOLIC\_DNA\_SENSING\_PATHWAY |  | 47 | 0.26 | 0.86 | 0.651 | 0.725 | 1.000 | 10503 | tags=36%, list=30%, signal=52% |
| 134 | KEGG\_FATTY\_ACID\_METABOLISM |  | 42 | 0.25 | 0.85 | 0.642 | 0.734 | 1.000 | 3425 | tags=10%, list=10%, signal=11% |
| 135 | KEGG\_RNA\_POLYMERASE |  | 27 | 0.25 | 0.79 | 0.792 | 0.829 | 1.000 | 26166 | tags=100%, list=75%, signal=405% |
| 136 | KEGG\_DRUG\_METABOLISM\_OTHER\_ENZYMES |  | 42 | 0.26 | 0.79 | 0.822 | 0.832 | 1.000 | 7646 | tags=24%, list=22%, signal=30% |
| 137 | KEGG\_GLYCOSAMINOGLYCAN\_DEGRADATION |  | 21 | 0.25 | 0.79 | 0.719 | 0.827 | 1.000 | 6409 | tags=19%, list=18%, signal=23% |
| 138 | KEGG\_OTHER\_GLYCAN\_DEGRADATION |  | 15 | 0.27 | 0.78 | 0.706 | 0.827 | 1.000 | 6753 | tags=20%, list=19%, signal=25% |
| 139 | KEGG\_PRIMARY\_IMMUNODEFICIENCY |  | 35 | 0.29 | 0.78 | 0.801 | 0.824 | 1.000 | 8711 | tags=29%, list=25%, signal=38% |
| 140 | KEGG\_ONE\_CARBON\_POOL\_BY\_FOLATE |  | 17 | 0.28 | 0.76 | 0.748 | 0.843 | 1.000 | 582 | tags=6%, list=2%, signal=6% |
| 141 | KEGG\_LINOLEIC\_ACID\_METABOLISM |  | 28 | 0.28 | 0.76 | 0.867 | 0.851 | 1.000 | 3650 | tags=18%, list=11%, signal=20% |
| 142 | KEGG\_SNARE\_INTERACTIONS\_IN\_VESICULAR\_TRANSPORT |  | 38 | 0.22 | 0.75 | 0.748 | 0.848 | 1.000 | 12291 | tags=32%, list=35%, signal=49% |
| 143 | KEGG\_NOD\_LIKE\_RECEPTOR\_SIGNALING\_PATHWAY |  | 61 | 0.24 | 0.75 | 0.693 | 0.849 | 1.000 | 8944 | tags=21%, list=26%, signal=29% |
| 144 | KEGG\_NICOTINATE\_AND\_NICOTINAMIDE\_METABOLISM |  | 24 | 0.27 | 0.73 | 0.771 | 0.872 | 1.000 | 7224 | tags=29%, list=21%, signal=37% |
| 145 | KEGG\_BASAL\_TRANSCRIPTION\_FACTORS |  | 33 | 0.18 | 0.73 | 0.777 | 0.868 | 1.000 | 13495 | tags=39%, list=39%, signal=64% |
| 146 | KEGG\_ALPHA\_LINOLENIC\_ACID\_METABOLISM |  | 18 | 0.30 | 0.72 | 0.811 | 0.869 | 1.000 | 2876 | tags=17%, list=8%, signal=18% |
| 147 | KEGG\_BASE\_EXCISION\_REPAIR |  | 33 | 0.23 | 0.70 | 0.732 | 0.894 | 1.000 | 13401 | tags=48%, list=39%, signal=79% |
| 148 | KEGG\_PEROXISOME |  | 78 | 0.18 | 0.69 | 0.866 | 0.896 | 1.000 | 8965 | tags=18%, list=26%, signal=24% |
| 149 | KEGG\_STARCH\_AND\_SUCROSE\_METABOLISM |  | 39 | 0.22 | 0.68 | 0.886 | 0.899 | 1.000 | 6980 | tags=23%, list=20%, signal=29% |
| 150 | KEGG\_RNA\_DEGRADATION |  | 51 | 0.13 | 0.67 | 0.947 | 0.911 | 1.000 | 30355 | tags=100%, list=87%, signal=793% |
| 151 | KEGG\_CYSTEINE\_AND\_METHIONINE\_METABOLISM |  | 34 | 0.14 | 0.65 | 0.935 | 0.921 | 1.000 | 12206 | tags=44%, list=35%, signal=68% |
| 152 | KEGG\_SELENOAMINO\_ACID\_METABOLISM |  | 24 | 0.21 | 0.62 | 0.887 | 0.943 | 1.000 | 5078 | tags=13%, list=15%, signal=15% |
| 153 | KEGG\_UBIQUITIN\_MEDIATED\_PROTEOLYSIS |  | 130 | 0.09 | 0.52 | 0.981 | 0.982 | 1.000 | 14789 | tags=39%, list=43%, signal=68% |
Table: Gene sets enriched in phenotype **h (8 samples) **[plain text format]****
